# A Successful Case of Transfusion of Blood

**Published:** 1880-12

**Authors:** 


### A SUCCESSFUL CASE OF TRANSFUSION OF BLOOD.

THE following case, which exhibits in a marked degree the beneficial effects of transfusion of blood, when performed in cases of impending death from excessive hemorrhage, is reported in the *New York Medical Journal*, for August, 1880, by Joseph W. Howe, M. D.

Mrs. B., aged twenty-two years, was delivered of a three months' fœtus, November 7th, 1879. From that date until November 11th she had repeated and profuse hemorrhages from the uterus. On the 10th the bleeding was continuous. Drs. Reynolds and Comstock, who were first called in, succeeded in controlling the hemorrhage, but not before the patient had reached the stage of collapse. They remained with her all night, endeavoring, with the ordinary means of stimulation, to rouse her, but without avail. She continued to sink in spite of everything.

On the morning of the 11th I was sent for. The patient was then completely pulseless and partially unconscious. The extremities were cold and clammy, and it was evident that unless some fresh blood were introduced death would soon supervene. She was so far gone, that I made up my mind not to spend any time in defibrinating the blood. I opened the median basilic vein in the right arm of the patient, and introduced the closed cannula of Colin's instrument, and after passing some warm water through the cylinder of the instrument, attached it to the cannula in the patient's arm. The median cephalic vein in the right arm of the donor was then opened, and the blood was allowed to flow directly into the cylinder without defibrination. When a sufficient quantity had been obtained, and while the blood was still flowing, I injected, without any difficulty, between seven and eight ounces. The whole operation did not occupy more than five minutes in its performance.

Within half an hour the pulse returned at the wrist, the voice became clear and distinct, and she asked for something to eat, saying that she felt stronger and better in every way. One of the medical gentlemen who had been with her all night, assisting in the attempts at resuscitation, and who left in the morning, believing that there was no hope of her recovery, came in an hour after the operation, and said it was "a perfect transformation scene,"—that he had no idea that a few ounces of blood could restore lost vitality so rapidly.

From that time on the patient continued to improve, and when I last heard from her she was in the enjoyment of good health and attending to her household duties without any discomfort whatever.

